# Severe Pediatric TBI Management in a Middle-Income Country and a High-Income Country: A Comparative Assessment of Two Centers

**DOI:** 10.3389/fsurg.2021.670546

**Published:** 2021-08-12

**Authors:** Jorge I. Arango, Laeth George, Dylan P. Griswold, Erica D. Johnson, Maria N. Suarez, Laura D. Caquimbo, Milton Molano, Raul A. Echeverri, Andres M. Rubiano, P. David Adelson

**Affiliations:** ^1^Department of Neurosurgery, Barrow Neurological Institute, Phoenix, AZ, United States; ^2^Phoenix Children's Hospital, Phoenix, AZ, United States; ^3^Department of Neurosurgery, College of Medicine Phoenix, University of Arizona, Phoenix, AZ, United States; ^4^Department of Neurosurgery, University of Cambridge, Cambridge, United Kingdom; ^5^School of Medicine, Stanford University, Stanford, CA, United States; ^6^School of Medicine, University of Pittsburgh, Pittsburgh, PA, United States; ^7^School of Medicine, South Colombian University, Neiva, Colombia; ^8^MEDITECH Foundation, Cali, Colombia; ^9^Neurological Surgery Service, Vallesalud Clinic, Cali, Colombia; ^10^INUB-MEDITECH Research Group, Neuroscience Institute, El Bosque University, Bogotá, Colombia

**Keywords:** traumatic brain injury, pediatric neurosurgery, global health, TBI, pediatric

## Abstract

**Background:** Traumatic brain injury (TBI) is a global public health issue with over 10 million deaths or hospitalizations each year. However, access to specialized care is dependent on institutional resources and public health policy. Phoenix Children's Hospital USA (PCH) and the Neiva University Hospital, Colombia (NUH) compared the management and outcomes of pediatric patients with severe TBI over 5 years to establish differences between outcomes of patients managed in countries of varying resources availability.

**Methods:** We conducted a retrospective review of individuals between 0 and 17 years of age, with a diagnosis of severe TBI and admitted to PCH and NUH between 2010 and 2015. Data collected included Glasgow coma scores, intensive care unit monitoring, and Glasgow outcome scores. Pearson Chi-square, Fisher exact, *T*-test, or Wilcoxon-rank sum test was used to compare outcomes.

**Results:** One hundred and one subjects met the inclusion criteria. NUH employed intracranial pressure monitoring less frequently than PCH (*p* = 0.000), but surgical decompression and subdural evacuation were higher at PCH (*p* = 0.031 and *p* = 0.003). Mortality rates were similar between the institutions (15% PCH, 17% NUH) as were functional outcomes (52% PCH, 54% NUH).

**Conclusions:** Differences between centers included time to specialized care and utilization of monitoring. No significant differences were evidenced in survival and the overall functional outcomes.

## Introduction

Traumatic brain injury (TBI) is an etiologically heterogeneous condition that represents a global public health issue with significant socioeconomic impact (1). According to the School of Public Health at Harvard, 57 million people have sought care for TBI at some point in their lives, and at least ten million of those events have been severe enough to result in death or require hospitalization (2). In the United States, pediatric TBI alone is estimated to have caused ~1,484 deaths; 17,930 hospitalizations; and 641,935 emergency department visits in 2013 (3). While epidemiological data on pediatric TBI in Latin America is limited, it is known that TBI-associated death rates are more than 75 per 100,000 in the continent, and these ascend to 125 per 100,000 in Colombia (4). TBI is the leading cause of death in children in Medellin, a major city in Colombia (5).

Guidelines for the acute medical management of severe TBI in infants, children, and adolescents were initially published in 2003 and most recently revised in 2012 by the Brain Trauma Foundation (6, 7). These guidelines provide information about intracranial pressure, perfusion, and oxygenation monitoring as well as recommendations for their management. The guidelines also address the use of imaging, seizure prophylaxis and management, sedation, pain management, temperature control, nutrition, and surgical intervention. Despite the beneficial effects of adherence to standardized approaches (8–13), there are a multitude of factors that may prevent clinicians or institutions from adhering to such guidelines. The evidence level of the proposed interventions, hospital infrastructure, and policy, provider training, staffing adequacy, equipment availability, and consumable supplies have previously been identified as some of those factors (14, 15).

Phoenix Children's Hospital (PCH) is a pediatric hospital ranked among the best in the United States for neurology and neurosurgery (16), and the Neiva University Hospital (NUH) is a tertiary care hospital and Level I trauma center in Neiva, Colombia (17). While both institutions are located in urban areas, one is in a high-income country and the other at a middle-income country (18). This is a socioeconomic factor that has shown to affect adherence to evidence-based care and effective decision-making, especially due to differences in resources (19). NUH is a public hospital with an important burden of general adult and pediatric patients coming from urban-rural areas of southern Colombia. Resources are limited, especially related to neuromonitoring devices and some medications that can be considered expensive. PCH is one of the largest private pediatric centers in the western region of the United States and has easy access to a variety of advanced resources for the care of pediatric patients. In this study, we compared the characteristics, management, and outcomes of children with severe TBI, admitted to PCH and NUH. Prehospital care systems in Phoenix are different than in Neiva due to less advanced resources for care during transport in the Colombian city. Emergency care and surgical care wards are similar with some variations in resource availability. Intensive care units (ICU) at PCH have more neuromonitoring resources than in NUH. We hypothesized that mortality could be larger in NUH when compared with the mortality at PCH.

## Methods

Our team performed a retrospective chart review of patients ages between 0 and 17 years, who presented to PCH, USA, and the NUH, Colombia with a diagnosis of severe TBI between July 1, 2010 and July 31, 2015. Records of patients with severe TBI were considered for analysis. For the purpose of this review, severe TBI is defined as a reported or evidential mechanical insult to the brain where the patient presents with Glasgow coma score (GCS) of 8 or less at admission or with GCS of 9 to 12 at admission, but with rapid deterioration within the first 24 h to GCS of 8 or less in the absence of underlying conditions that may influence their neurological function.

Collected data included demographic characteristics of the patients, information about the injury event, prehospital and hospital management, and imaging findings and discharge status. Record selection and data extraction were performed simultaneously. At PCH, case identification was accomplished by querying the electronic medical records system for diagnostic codes corresponding to TBI and subsequent exploration of ancillary database systems from the emergency department, surgery, anesthesia, and the ICU. The NUH research team identified subjects by searching the electronic medical record of the hospital pediatric ICU for diagnostic codes corresponding to TBI. Subjects were then screened for eligibility, and their information was extracted by medical students utilizing a data collection form.

Identifying information about patients was removed from both datasets and compiled for analysis after data extraction was completed. Data analysis was performed by the PCH group using IBM SPSS Statistics software (Chicago, IL). A descriptive analysis was performed to visualize population and site characteristics. Pearson Chi-square, Fisher exact, *T*-test, Kruskal–Wallis, or Wilcoxon-rank sum test was used to compare interinstitutional patient characteristics, management, and outcomes. Univariate linear regression was used to assess potential associations between associated injuries and radiological findings and hospital outcomes.

## Results

Sixty-six patients (34 males, 32 females) from PCH and 35 patients (24 male, 11 female) from NUH met the criteria for inclusion in the study. The mean age at PCH and Neiva was 7.7 and 6.6 years, respectively. GCS at presentation was similar between the groups (PCH 5.42 and NUH 5.46). Motor vehicle accidents were the primary cause of injury at both institutions (PCH 51.5% and NUH 68.6%) (Table 1). Most patients were transferred from the scene of the injury or home to PCH (68.0%). Transfers from a different hospital were the most common source for patients receiving care at NUH (80%). Subsequently, the overall time from injury to arrival at the treating hospital was significantly longer at NUH (NUH 5 h and 31 min, PCH 1 h and 51 min, *p* = 0.025) (Table 2). Most patients received advance life support before their arrival to the respective treatment centers. Both resuscitation efforts and ventilatory support were more commonly provided at NUH [NUH 46 (97%), PCH 9 (68%), respectively].

**Table 1:** Cause and type of injuries.

**Cause of injury**	**PCH *n* (%)**	**NUH *n* (%)**
Accidental falls	21 (31.8)	6 (17.1)
Assault	6 (9.1)	2 (5.7)
Motor vehicle accident	34 (51.5)	24 (68.6)
Self-inflicted	3 (4.5)	0 (0.0)
Other	2 (3.0)	3 (8.6)
**Type of injury**
Closed	62 (93.9)	30 (85.7)
Penetrating	3 (4.5)	4 (11.4)
Crush	1 (1.5)	1 (2.9)

**Table 2:** Time from injury to arrival by patient origin.

**Patient origin**	**PCH (h:m)**	**NUH (h:m)**
Scene	1:45	3:52
Home	2:30	6:20
Other hospital	1:18	6:53
Total	1:51	5:31

Traumatic brain injury was often associated with injuries to other anatomical locations (NUH 93%, PCH 68%; Table 3). The most affected areas were upper and lower extremities (NUH 37%, PCH 42%), face (NUH 34%, PCH 15%), and thorax (NUH 17%, PCH 18%). No association was observed between the number or type of associated injuries and hospital outcomes, and hospital and length of stay in the ICU. The lack of association was independent of location (PCH, NUH). Computer tomography (CT) was performed at admission in all patients from both institutions. Skull fractures were the most common findings among patients from both locations, followed by subdural hematomas and contusions at PCH and by diffuse axonal injury and midline shift at NUH. Notable differences were observed in the reported incidence of subdural hematomas and diffuse axonal injuries (Table 4). An association was observed between the imaging presence of subarachnoid hemorrhages, intraparenchymal hemorrhages, and midline with the length of the hospital stay of the patients (*p* = 0.02, 0.02, and 0.43, respectively).

**Table 3:** Number of additional non-TBI injuries.

**Number**	**PCH *n* (%)**	**NUH *n* (%)**
None	21 (32)	2 (7)
1	14 (21)	15 (56)
2	11 (17)	6 (22)
3	20 (30)	4 (15)

**Table 4:** CT findings.

**CT finding**	**PCH *n* (%)**	**NUH *n* (%)**	**Significance**
Skull fracture	43 (65)	26 (74)	0.24
Subdural hematoma	36 (55)	4 (11)	<0.01
Contusion	34(51)	9(26)	0.01
Sub-arachnoid hemorrhage	28 (42)	7 (20)	0.02
Cisterns partial or full collapse	27(41)	4(11)	<0.01
Intra-parenchymal hematoma	22 (33)	5 (14)	0.03
Midline shift	20 (30)	10 (29)	0.52
Intra-ventricular hemorrhage	13 (20)	0 (0)	<0.01
Diffuse axonal injury	10 (15)	16 (46)	<0.01
Epidural hematoma	7(11)	8(23)	0.09

Intracranial pressure (ICP) monitoring was used more often at PCH (PCH 68.0%, NUH 14.0%). This trend was also observed with other invasive monitoring modalities such as intraparenchymal temperature and brain tissue partial pressure neuromonitoring. NUH favored pharmacological management for ICP control (NUH 94%, PCH 70%), whereas PCH opted for surgical management through external ventricular drain (EVD) placement and decompressive craniectomy (Table 5). Drainage of intracranial fluid collections (epidural, subdural, and intraparenchymal) was performed at a higher rate at PCH (PCH 32%, NUH 11%).

**Table 5:** Invasive monitoring and ICP management.

**Monitoring modalities**	**PCH *n* (%)**	**NUH *n* (%)**
Intracranial pressure	45 (68)	5 (14)
Tissular oxygen pressure	16 (24)	0 (0)
Intraparenchymal temperature	13 (20)	0 (0)
**Pharmacologic**
Hypertonic saline + Mannitol	13 (20)	18 (51)
Hypertonic saline	19 (29)	10 (26)
Mannitol	14 (21)	5 (14)
**Surgical**
EVD placement	37 (56)	0 (0)
Decompressive craniectomy	21 (32)	3 (9)
Unilateral	17 (26)	2 (6)
Bifrontal	4 (6)	1 (3)

Seizure prophylaxis was widely used in both institutions, but the medications utilized varied widely. At PCH, either fosphenytoin or levetiracetam was used in 74% of the patients, either as monotherapy or in combination. At NUH, phenytoin was used almost exclusively (86%). Seizure reporting in the early (≤7 days) and late (>7 days) periods was similar for both institutions. PCH reported early seizures in 29.0% of patients and late seizures in 6.0%, whereas NUH saw a rate of 26.0 and 6.0%, respectively. Levetiracetam was the medication of choice for seizure management at PCH, and it was used as monotherapy in 63.0% of the patients who developed seizure activity and in combination with fosphenytoin in 10% of the cases. Fosphenytoin and phenobarbital were periodically used as monotherapy. Carbamazepine, phenytoin, and valproic acid were equally used for seizure management at NUH.

While the length of stay varied widely within institutions, no significant differences were observed between the mean length of stay at the hospital or in the ICU. Functional outcomes were assessed using the Glasgow outcome scale (GOS), and despite evidencing disparity in absolute numbers and distribution, the difference failed to attain statistical significance. Outcomes were further grouped as dead, severely disabled, or with low disability. This simplified outcome scale was chosen to limit the potential inaccuracies of excessive stratification; yet the overall behavior of the sample remained similar and no statistical difference was observed (Table 6).

**Table 6:** Functional and institutional outcomes.

**Length of stay (days)**	**PCH μ (SD)**	**NUH μ (SD)**	**Significance**
Hospital	33 (44)	28 (34)	0.32^*^
Intensive care unit	12 (13)	13 (25)	0.09^*^
**Glasgow outcome scale**	**PCH** ***n*** **(%)**	**NUH** ***n*** **(%)**	**Significance**
1. Death	10 (15)	6 (19)	0.26^‡^
2. Persistent vegetative state	3 (5)	0 (0)	
3. Severe disability	18 (28)	7 (22)	
4. Moderate disability	16 (25)	4 (12)	
5. Low disability	18 (28)	15 (47)	
**Outcome categories**
Dead	10 (16)	6 (19)	0.56^‡^
Severe disability	21 (32)	7 (22)	
Low disability	34 (52)	19 (59)	

* *P-value from Kruskal-Wallis test*.

‡ *P-value from Fisher-exact test*.

## Discussion

The results of this comparison reveal the experiences of two different institutions at managing severe pediatric TBI in populations that were demonstrated to be homogeneous in age, gender, and injury severity. Considering the geographic location of these institutions, one at a high income, developed country and the other one at a middle income, developing county, assumptions can be drawn about resource availability and access to care. Despite motor vehicle accidents being the most common mechanism of injury in both populations, transport from the scene to the treating hospital was predominant among patients treated at PCH. Transport from the scene to the treating hospital was seen in only 20% of the cases treated at NUH. This difference would likely explain the longer mean time from injury to arrival at the treatment facility observed among the NUH cohort. However, this delay in access to specialized care does not necessarily translate into treatment delay considering that most patients treated at NUH were transferred from other hospitals, and advance life support measures were in place upon arrival more often than at PCH. Pre-hospital care has previously been considered as a potential confounder of clinical outcomes in studies comparing TBI management between developed and developing countries. Gupta et al. found similar transport times between a hospital in New Delhi and a hospital in Seattle, but because of similar limitations as the ones in our study, a direct relationship between pre-hospital care and outcomes was not drawn (20).

The presence of associated injuries in locations other than the head was more often seen in the NUH cohort. However, neither the presence of injuries at specific locations nor the number of areas compromised demonstrated to have any impact on hospital or ICU length of stay. Skull fractures were the most common imaging finding, and no difference was observed in the frequency at which it was reported between cohorts. With the exemption of diffuse axonal injury and epidural hematomas, most radiological findings were most reported among the PCH cohort. Whether this difference is due to the absence of findings, the quality of the scan, or the reporting practices at each institution warrants further exploration.

Significant differences were observed in invasive monitoring and ICP management. Patients at NUH were treated more conservatively with minimal use of invasive monitoring and predominance of pharmacologic management. In contrast, PCH frequently employed invasive monitoring for ICP and used other invasive monitoring modalities in the most severe cases. EVD placement and cranial decompression were used more often, while pharmaceutical ICP management was used less often.

Seizure prophylaxis was widely used in both institutions. There was substantial variability in the medications used between institutions, but the incidence of seizure was vastly the same among cohorts.

No significant differences were observed on any of the variables selected as outcomes. Length of stay remained within 10% of the overall mean for hospital and ICU stay at both institutions. NUH patients displayed a higher mortality rate, but the mortality rates observed at both institutions seemed to be at the lower end of those reported in similar populations (21). Survivors from the NUH group also presented lower disability in GOS at discharge than patients at PCH. However, neither of these differences attained statistical significance and became more homogeneous when grouped as either dead, having a severe disability, or a mild disability.

The institutions selected for the comparison were intended to provide a surrogate view of the countries they belong to and their socio-economic environments. While institutional policies are usually a significant driver for care, management characteristics can indicate resource availability and utilization. Thus, the increased use of invasive monitoring and management at PCH is suggestive of higher resource utilization when compared to NUH. Interestingly and contrary to previously described by other authors, neither advance monitoring nor early decompression surgery seemed to have an effect in mortality rates or short-term neurological outcomes (22).

Limitations of this study begin with its methodology as a retrospective chart review. Thus, the study lacks the accuracy and reliability a prospective cohort study would offer. As a result, the study is inherently limited by coding mechanisms, record completeness, and information availability. Furthermore, the relatively small sample size may have affected the lack of statistical significance in the heterogeneous areas contributing to the mortality rate that we hypothesized would be statistically significant.

## Conclusion

Despite the heterogeneous character of severe TBI and differences in the time from injury to specialized treatment, radiologic findings, monitoring, and surgical management, we found no significant differences in functional or hospital outcomes between two similar pediatric cohorts of patients treated for severe TBI at PCH, in a high-income country, and NUH, in a middle-income country. However, given the retrospective character and the relatively small sample size of our study, we call for cautiousness when interpreting our results and rather move the focus toward the disparities in clinical resources between developing countries and developed countries in which most TBI research is conducted and guidelines are written, and highlight the need for resource consideration when disseminating practice guidelines.

## Data Availability Statement

The original contributions presented in the study are included in the article/supplementary material, further inquiries can be directed to the corresponding author/s.

## Author Contributions

All authors listed have made a substantial, direct and intellectual contribution to the work, and approved it for publication.

## Conflict of Interest

The authors declare that the research was conducted in the absence of any commercial or financial relationships that could be construed as a potential conflict of interest.

## Publisher's Note

All claims expressed in this article are solely those of the authors and do not necessarily represent those of their affiliated organizations, or those of the publisher, the editors and the reviewers. Any product that may be evaluated in this article, or claim that may be made by its manufacturer, is not guaranteed or endorsed by the publisher.
